# Beyond the Four Pillars: A Risk-Targeted Framework for Vericiguat—A Narrative Review

**DOI:** 10.3390/jcm15145749

**Published:** 2026-07-22

**Authors:** Jacek Kubica, Aldona Kubica, Robert Gajda, Ewa Laskowska, Julia M. Umińska, Jakub Ratajczak, Piotr Niezgoda, Natalia Mrzywka, Łukasz Szarpak, Eliano P. Navarese

**Affiliations:** 1 Collegium Medicum, Nicolaus Copernicus University, 85-094 Bydgoszcz, Poland; jkubica@cm.umk.pl (J.K.); akubica@cm.umk.pl (A.K.); ewa.laskowska@cm.umk.pl (E.L.); julia.uminska@cm.umk.pl (J.M.U.); piotr.niezgoda@cm.umk.pl (P.N.); natalia.mrzywka@onet.eu (N.M.); elianonavarese@gmail.com (E.P.N.); 2 Modern Medical Technologies Center, 87-100 Torun, Poland; gajda@gajdamed.pl; 3 Gajda-Med District Hospital, 06-102 Pultusk, Poland; 4 Institute of Medical Sciences, The John Paul II Catholic University of Lublin, 20-950 Lublin, Poland; lukaszszarpak@gmail.com; 5 Department of Life and Health Sciences, Link Campus University, 00165 Rome, Italy

**Keywords:** heart failure, vericiguat, soluble guanylate cyclase, guideline-directed medical therapy

## Abstract

Heart failure (HF) remains a major global health burden despite substantial therapeutic advances. Vericiguat, an oral soluble guanylate cyclase (sGC) stimulator, represents a distinct pharmacological strategy targeting impaired nitric oxide–sGC–cyclic guanosine monophosphate (NO–sGC–cGMP) signaling, a pathway closely linked to endothelial dysfunction, vascular stiffness, myocardial fibrosis, and adverse remodeling. This narrative review synthesizes the current evidence on the efficacy, safety, and clinical positioning of vericiguat across the heart failure spectrum, with a particular focus on worsening heart failure with reduced ejection fraction (HFrEF), while integrating recent trial data, updated guideline recommendations, and emerging real-world evidence to define its contemporary role in clinical practice. Randomized trials, subgroup analyses, and contemporary meta-analyses indicate that vericiguat provides a modest but clinically relevant reduction in HF-related outcomes, especially in high-risk patients with recent decompensation. However, its overall effect appears smaller than that of foundational therapies such as angiotensin receptor–neprilysin inhibitors and sodium–glucose cotransporter 2 inhibitors. Recently published data from the VICTOR program further refine the role of vericiguat in compensated outpatients with chronic HFrEF, showing a neutral primary outcome but providing supportive hypothesis-generating signals across a broader risk continuum while confirming a favorable safety profile. Overall, vericiguat should be regarded not as a replacement for cornerstone therapy but as a mechanistically complementary, risk-targeted adjunct for selected patients with persistent residual risk despite guideline-directed medical therapy.

## 1. Introduction

Despite major therapeutic progress, heart failure (HF) continues to be associated with high morbidity, recurrent hospitalizations, impaired quality of life, and premature mortality. Contemporary management of heart failure with reduced ejection fraction (HFrEF) is built on quadruple therapy including renin–angiotensin system inhibition, preferably with angiotensin receptor–neprilysin inhibitors (ARNI), β-blockers, mineralocorticoid receptor antagonists (MRA), and sodium–glucose cotransporter 2 (SGLT2) inhibitors. Yet even with optimal implementation of these therapies, a substantial proportion of patients remain symptomatic or experience recurrent worsening events, underscoring the persistence of residual pathophysiological risk despite modern guideline-directed medical therapy (GDMT) [1,2,3,4,5].

Vericiguat is an oral stimulator of soluble guanylate cyclase (sGC) that enhances cyclic guanosine monophosphate (cGMP) production by directly stimulating the enzyme and sensitizing it to endogenous nitric oxide. This mechanism addresses impaired nitric oxide signaling, a central feature of HF biology that is not directly corrected by standard foundational therapies [1,2,3]. Following the biological signal observed in SOCRATES-REDUCED and the clinical outcome benefit demonstrated in VICTORIA, vericiguat emerged as a candidate therapy for patients with recent worsening HFrEF [1,3]. More recently, the VICTOR program has expanded the evidence base and renewed interest in the role of sGC stimulation across a broader chronic HFrEF risk continuum [6,7,8,9,10,11]. Although several recent reviews have summarized the clinical evidence supporting vericiguat, the publication of the VICTOR program and its subsequent analyses has substantially expanded the available evidence base. Moreover, the increasing emphasis on residual risk, individualized treatment strategies, and integration of emerging therapies into contemporary guideline-directed medical therapy warrants a reappraisal of the clinical positioning of vericiguat. Therefore, this review aims not only to summarize current evidence but also to provide a practical, risk-oriented framework for identifying patients most likely to benefit from vericiguat in routine clinical practice.

The aim of this narrative review is to summarize the mechanistic basis, efficacy, safety, and contemporary positioning of vericiguat, with particular emphasis on worsening HF and its place within increasingly personalized, layered treatment strategies.

## 2. Methods

This narrative review was conducted to provide a comprehensive overview of the mechanistic rationale, clinical efficacy, safety, and current clinical positioning of vericiguat in patients with heart failure, with particular emphasis on worsening heart failure with reduced ejection fraction (HFrEF).

A focused literature search was performed in the PubMed/MEDLINE database to identify relevant publications available from database inception through 1 May 2026. The search strategy combined Medical Subject Headings (MeSH) and free-text terms including “vericiguat”, “soluble guanylate cyclase”, “sGC”, “cyclic guanosine monophosphate”, “cGMP”, “heart failure”, “heart failure with reduced ejection fraction”, “HFrEF”, “heart failure with preserved ejection fraction”, “HFpEF”, “worsening heart failure”, “VICTORIA”, “VICTOR”, and “SOCRATES”.

Only articles published in English were considered. Eligible publications included randomized controlled trials, pooled and subgroup analyses, systematic reviews, meta-analyses, pharmacokinetic and pharmacodynamic studies, and relevant mechanistic investigations. Conference abstracts, editorials without original scientific content, duplicate publications, and studies not directly related to vericiguat in heart failure were excluded.

The electronic search identified approximately 493 records. After removal of duplicates and screening of titles and abstracts, approximately 169 articles underwent full-text assessment. Ultimately, 49 publications judged to be the most methodologically robust and clinically relevant were included in the review. Additional publications were identified through manual screening of reference lists of key clinical trials, recent reviews, and contemporary guideline-related publications.

As this manuscript was designed as a narrative review, no formal systematic review methodology, PRISMA reporting framework, or quantitative assessment of study quality was applied. Instead, studies were selected on the basis of methodological quality, clinical relevance, scientific impact, and their contribution to the contemporary understanding of the role of vericiguat in heart failure management.

The AI-assisted tool (ChatGPT 5, Open AI, San Francisco, CA, USA) was used for graphical rendering of the figures, but the conceptual design and scientific content were created by the authors.

## 3. Mechanism of Action and Rationale

The nitric oxide–soluble guanylate cyclase–cGMP (NO–sGC–cGMP) pathway is a central regulator of cardiovascular homeostasis. Under physiological conditions, endothelial-derived nitric oxide activates sGC in vascular smooth muscle cells and cardiomyocytes, increasing intracellular cGMP. This second messenger promotes vasorelaxation, improves myocardial relaxation, and exerts anti-hypertrophic, anti-inflammatory, and anti-fibrotic effects through downstream signaling pathways, including activation of protein kinase G [1,2,3]. In this way, intact cGMP signaling supports vascular compliance, limits myocardial stiffness, and attenuates maladaptive ventricular remodeling.

In HF, this protective axis becomes profoundly disturbed. Endothelial dysfunction, oxidative stress, neurohormonal activation, systemic inflammation, and recurrent congestion reduce nitric oxide bioavailability and impair sGC responsiveness [1,3,12]. Reactive oxygen species may further alter the redox state of sGC, rendering the enzyme less responsive to endogenous nitric oxide. The consequence is reduced cGMP generation and a loss of vasodilatory, antifibrotic, and anti-remodeling signaling. This pathobiological shift contributes to vasoconstriction, impaired ventricular relaxation, myocardial fibrosis, elevated filling pressures, and progressive structural deterioration of the failing heart [1,2,3,12].

**Figure 1:**
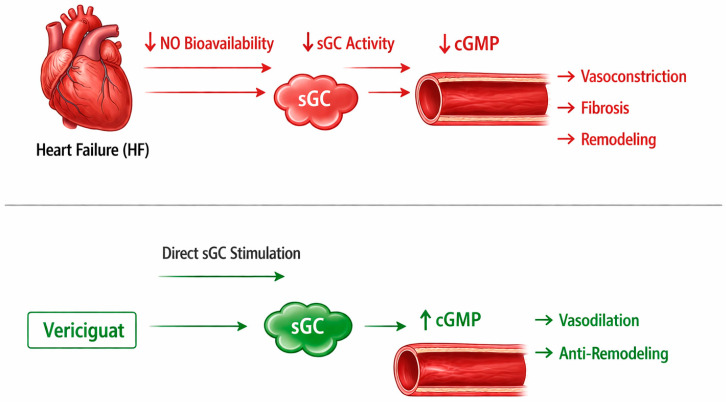
Mechanistic and clinical positioning of vericiguat in worsening heart failure. Heart failure is associated with endothelial dysfunction, oxidative stress, neurohormonal activation, and reduced nitric oxide bioavailability, leading to impaired soluble guanylate cyclase activity and reduced cGMP generation. This results in vasoconstriction, myocardial fibrosis, adverse remodeling, and progressive hemodynamic deterioration. Vericiguat directly stimulates soluble guanylate cyclase and sensitizes the enzyme to endogenous nitric oxide, thereby restoring cGMP signaling. The conceptual design and scientific content were created by the authors, and AI-assisted tools (ChatGPT 5, Open AI, San Francisco, CA, USA) were used solely for graphical rendering.

The rationale for vericiguat is therefore biologically compelling. Unlike therapies that primarily modulate neurohormonal pathways or sodium–glucose handling, vericiguat directly targets impaired sGC–cGMP signaling (Figure 1).

## 4. Clinical Evidence

In SOCRATES-REDUCED, vericiguat was associated with favorable reductions in natriuretic peptide levels in patients with worsening chronic HFrEF [1]. Subsequent pharmacokinetic and pharmacodynamic studies demonstrated a predictable interaction profile and supported co-administration with commonly used cardiovascular therapies [2]. These early findings provided a coherent mechanistic rationale for sGC stimulation, which was subsequently evaluated in large-scale clinical trials. Importantly, this rationale is biologically most compelling in the setting of worsening HF, where endothelial dysfunction, repeated hemodynamic stress, and neurohormonal overactivation are particularly pronounced. In this setting, conventional GDMT, although highly effective, may not fully restore impaired NO-sGC–cGMP signaling. Vericiguat may therefore address a biologically plausible therapeutic gap, particularly in patients whose clinical instability reflects persistent residual risk despite otherwise appropriate treatment [3,12,13].

### 4.1. Efficacy in Heart Failure with Reduced Ejection Fraction

The efficacy of vericiguat in HFrEF is best established in the setting of worsening HF. In the VICTORIA trial, vericiguat reduced the composite endpoint of cardiovascular death or first HF hospitalization in patients with chronic HFrEF and recent worsening HF, thereby defining a clinically meaningful role for sGC stimulation in this particularly vulnerable population [3]. The primary composite endpoint occurred in 35.5% of patients receiving vericiguat compared with 38.5% receiving placebo (HR 0.90, 95% CI 0.82–0.98), corresponding to an absolute risk reduction of approximately 3.0% over the median follow-up and a number needed to treat of approximately 33. Importantly, the observed benefit was driven predominantly by a reduction in HF hospitalizations, whereas cardiovascular mortality alone was not significantly reduced [3]. This finding is particularly relevant given that VICTORIA enrolled a distinctly higher-risk cohort than many landmark HFrEF trials, with markedly elevated natriuretic peptide concentrations and recent decompensation.

Subsequent analyses refined the interpretation of this treatment effect. Baseline N-terminal prohormone of brain natriuretic peptide (NT-proBNP) appeared important for contextualizing efficacy, with the most advanced biological risk associated with attenuation of relative benefit [12,13]. Clinical response also varied according to the index worsening event, suggesting that both the trajectory and recency of decompensation influence the expected yield of therapy [14]. Analyses of recurrent hospitalizations further indicated that vericiguat may reduce the burden of repeated HF admissions, an increasingly relevant endpoint in contemporary HF trials [15]. Additional subgroup analyses suggested that the benefit of vericiguat is broadly preserved across clinically relevant patient subsets, including those with diabetes, atrial fibrillation, and impaired renal function [16,17,18].

Meta-analytic evidence has generally confirmed that vericiguat provides a modest but genuine benefit in HFrEF. Conventional pairwise meta-analyses showed improvement in composite HF outcomes with a favorable safety profile [19,20]. A broader updated meta-analysis across the HF spectrum reached similar conclusions while emphasizing that the strength of evidence is greatest in HFrEF rather than heart failure with preserved ejection fraction (HFpEF) [21]. In addition, a network meta-analysis focused on worsening HF identified vericiguat as one of the novel therapies capable of reducing rehospitalization risk in this high-risk setting [22]. However, the magnitude and clinical relevance of this benefit require careful contextualization.

Comparative analyses consistently indicate that the magnitude of benefit associated with vericiguat is smaller than that achieved with foundational therapies. An updated network meta-analysis of pharmacological treatment for HFrEF ranked ARNI and SGLT2 inhibitors among the most effective strategies, whereas vericiguat appeared to provide incremental benefit rather than a transformative effect [23]. Similar conclusions were reached in analyses evaluating optimal combinations of HFrEF therapies and in direct comparative network meta-analyses against sacubitril/valsartan [24,25]. Subgroup-oriented syntheses further support the view that vericiguat performs best in patients with greater baseline risk or recent clinical instability [26,27].

The VICTOR trial expanded the evidence base by evaluating vericiguat in ambulatory patients with chronic HFrEF without recent worsening under contemporary GDMT [6]. The primary composite endpoint of cardiovascular death or HF hospitalization occurred in 18.0% of patients receiving vericiguat and 19.1% receiving placebo (HR 0.93, 95% CI 0.83–1.04; *p* = 0.22), and therefore the overall trial should be regarded as neutral with respect to its primary efficacy objective [7]. Although cardiovascular mortality (9.6% vs. 11.3%; HR 0.83, 95% CI 0.71–0.97) and all-cause mortality (12.3% vs. 14.4%; HR 0.84, 95% CI 0.74–0.97) were numerically lower in the vericiguat group, these analyses were based on prespecified secondary endpoints and should therefore be considered hypothesis-generating rather than confirmatory [7,11]. Consequently, the VICTOR findings should be interpreted as refining the understanding of potential treatment effects across the chronic HFrEF risk spectrum rather than expanding the established clinical indication for vericiguat.

Additional analyses suggested acceptable blood pressure tolerance and consistent treatment effects across a range of baseline hemodynamic profiles, without clear safety concerns [8]. An individual participant data analysis combining VICTORIA and VICTOR suggested that the potential benefit of vericiguat may extend across a broader risk continuum, although the magnitude of clinical benefit remains closely related to baseline risk [9]. Complementary analyses from the VICTOR trial suggested possible reductions in total HF events and provided supportive, hypothesis-generating signals regarding mortality [10,11].

Taken together, these findings broaden the understanding of vericiguat across different clinical risk profiles; however, the strongest evidence supporting its use remains confined to patients with recent worsening HFrEF, consistent with the VICTORIA trial and current guideline recommendations (Table 1).

### 4.2. Efficacy Across the Spectrum of Ejection Fraction

Evidence for vericiguat beyond classic HFrEF is less compelling. In SOCRATES-PRESERVED, vericiguat generated biologically interesting signals in patients with worsening HF and preserved ejection fraction, but the results were insufficient to establish a clear clinical role [28]. The subsequent VITALITY-HFpEF trial did not demonstrate a significant improvement in quality of life compared with placebo, thereby limiting enthusiasm for routine use in HFpEF [29].

More recently, a patient-level pooled meta-analysis of VITALITY-HFpEF and VICTORIA suggested that vericiguat may exert effects across a range of ejection fractions, although the magnitude of benefit appears to diminish as ejection fraction rises [30]. Updated meta-analytic evidence similarly supports a more favorable profile in reduced than in preserved ejection fraction [21]. At present, therefore, the clinical role of vericiguat remains centered on HFrEF, whereas its place in heart failure with mildly reduced ejection fraction (HFmrEF) and HFpEF remains uncertain and investigational. This evidence gap largely reflects the absence of dedicated randomized clinical trials specifically enrolling patients with HFmrEF. Future studies should determine whether specific HFmrEF phenotypes, particularly those sharing biological characteristics with HFrEF, may benefit from soluble guanylate cyclase stimulation.

### 4.3. Comparative Effectiveness in Contemporary HF Therapy

The key question is not whether vericiguat works but where it fits within the modern HF treatment hierarchy. Comparative evidence strongly suggests that vericiguat should not be viewed as an alternative to foundational therapies. ARNI and SGLT2 inhibitors consistently produce larger reductions in mortality and HF hospitalization and therefore remain central to first-line treatment strategies [23,24,25,26,27]. Vericiguat is more appropriately understood as a second-line or add-on therapy, particularly in patients with persistent symptoms, recurrent HF events, or other markers of residual instability despite optimized GDMT.

Several observations reinforce this positioning. First, analyses of background therapy in VICTORIA showed that vericiguat was tested on top of increasingly contemporary HF treatment, underscoring its role as an adjunct rather than a substitute [31]. Second, efficacy appeared generally preserved in patients receiving sacubitril/valsartan, suggesting that the effect of vericiguat is not merely redundant with neprilysin inhibition [32]. Third, modeling exercises projecting VICTORIA data onto PARADIGM-HF and DAPA-HF-type populations indicate that the anticipated benefit of vericiguat depends heavily on baseline event risk, with the greatest benefit expected in patients with the highest residual risk after standard therapy [33]. This risk-based therapeutic positioning is consistent with recent comparative evidence demonstrating that the greatest absolute benefit of adjunctive therapies is achieved in patients with the highest baseline clinical risk, whereas foundational therapies continue to represent the cornerstone of HFrEF management [26].

### 4.4. Safety and Tolerability

Across randomized trials and meta-analyses, vericiguat has demonstrated a favorable and generally manageable safety profile. The principal adverse events of interest are hypotension and anemia, but treatment discontinuation due to intolerance has generally been infrequent [19,20]. The blood pressure analysis from VICTOR is particularly informative, as it supports the feasibility of vericiguat even in patients in whom further hemodynamic compromise could be a concern, provided that therapy is introduced thoughtfully and monitored appropriately [8].

Several dedicated studies further refine this safety profile. Phase I and Phase Ib interaction studies showed acceptable pharmacokinetic and hemodynamic compatibility with nitrates and other cardiovascular drugs, while also clarifying practical precautions [2,34,35]. A dedicated QTc study did not identify a clinically meaningful proarrhythmic signal [36]. Trial-level analyses in patients with chronic coronary syndromes similarly support cardiovascular tolerability [36]. Renal subgroup analyses from VICTORIA suggest that the treatment effect is preserved across categories of kidney function without a major renal safety penalty [18]. Likewise, efficacy and safety appear similar in patients with atrial fibrillation and in those with diabetes [16,17].

These observations are clinically relevant because the patients most likely to receive vericiguat are often older, multimorbid, and already exposed to complex multidrug regimens. In such populations, the utility of an incremental therapy depends not only on efficacy but also on practical tolerability and compatibility with existing treatment.

### 4.5. Clinical Positioning of Vericiguat

Taken together, the available evidence supports a niche but clinically meaningful role for vericiguat. It is best positioned in patients with chronic symptomatic HFrEF who remain at elevated risk despite appropriate use of foundational GDMT, especially those with recent worsening HF, recurrent hospitalization, persistent congestion, elevated natriuretic peptides, or other markers of instability [3,9,10,11,22]. Vericiguat therefore aligns with a risk-based treatment philosophy: the greater the residual risk, the stronger the rationale for incremental pathway-specific intervention.

This concept is increasingly relevant in an era in which the challenge is no longer simply initiating HF therapy but determining what should be added, in whom, and when. Vericiguat may be particularly attractive in patients who remain vulnerable despite ARNI, β-blocker, MRA, and SGLT2 inhibitor therapy and in whom the biological signature suggests persistent hemodynamic and endothelial stress. Conversely, current evidence does not support routine early use in stable, lower-risk patients already doing well on foundational therapy.

Health-economic analyses indicate that vericiguat is associated with favorable cost-effectiveness when used in patients with worsening HFrEF following optimized GDMT. The economic benefit is driven predominantly by reductions in heart failure hospitalizations, resulting in additional quality-adjusted life-years (QALYs) at incremental cost-effectiveness ratios considered acceptable in contemporary health-economic evaluations. Comparable findings have been reported in analyses based on the US Medicare perspective [37,38].

### 4.6. Practical Considerations for Vericiguat Initiation and Monitoring

The clinical use of vericiguat requires careful patient selection, appropriate background optimization of GDMT, and structured clinical monitoring to maximize benefit while minimizing the risk of hypotension, anemia, and treatment intolerance. A practical approach to vericiguat initiation and monitoring is summarized in Table 2.

In routine practice, vericiguat is most appropriately considered in patients with chronic symptomatic HFrEF who remain at high risk despite optimized GDMT. This includes patients recently hospitalized for HF, those requiring intravenous diuretics, and those with recurrent decompensation, persistent congestion, elevated natriuretic peptides, or other markers of residual instability.

Before initiation, patients should be clinically stabilized and assessed for blood pressure, volume status, renal function, hemoglobin concentration, and overall tolerability of background therapy. Vericiguat is usually started at 2.5 mg once daily and gradually increased to 5 mg and then 10 mg once daily as tolerated. Early follow-up should focus primarily on blood pressure, symptoms of hypotension, congestion status, and treatment tolerance, with additional attention to hemoglobin in patients at risk of anemia.

Within contemporary HFrEF therapy, vericiguat should be viewed as a mechanistically complementary, risk-targeted adjunct rather than an alternative to foundational treatment. Its greatest clinical value appears to lie in patients who continue to experience worsening HF or remain clinically vulnerable despite appropriate use of ARNI/angiotensin-converting-enzyme inhibitors (ACEi)/angiotensin II receptor blockers (ARB), beta-blockers, MRA, and SGLT2 inhibitors.

### 4.7. Positioning Vericiguat in Contemporary Heart Failure Management: From Guidelines to a Risk-Targeted Framework

Current international heart failure guidelines position vericiguat as an adjunctive therapy for selected patients with symptomatic HFrEF who remain at high risk despite optimized guideline-directed medical therapy (GDMT), particularly following a recent worsening heart failure event [3,21,22,23,24,25,26,27,39,40,41]. Similarly, current regulatory approvals restrict its use to patients with symptomatic chronic HFrEF after a recent episode of decompensation requiring hospitalization or intravenous diuretic therapy [3].

These recommendations are primarily based on the VICTORIA trial, which established the clinical benefit of vericiguat in patients with worsening HFrEF [3]. More recently, the VICTOR program and subsequent patient-level pooled analyses have expanded the evidence base by exploring the role of vericiguat across a broader spectrum of ambulatory patients with chronic HFrEF [7,8,9,10,11]. Importantly, these findings should not be interpreted as extending the current regulatory indication but rather as refining the identification of patients who may derive the greatest incremental benefit from soluble guanylate cyclase stimulation [7,8,9,10,11].

The present review integrates mechanistic insights, pivotal randomized clinical trials, contemporary guideline recommendations, regulatory positioning, recent meta-analyses, and the latest evidence from the VICTOR program into a unified clinical framework [3,7,8,9,10,11,21,22,23,24,25,26,27]. We propose that vericiguat should be viewed as a risk-targeted adjunctive therapy for patients who continue to exhibit evidence of persistent biological and clinical vulnerability despite optimized foundational GDMT (Table 3). This framework emphasizes treatment escalation according to residual risk rather than chronological sequencing alone and may facilitate more individualized therapeutic decision-making in contemporary HFrEF management. As such, it complements existing guideline recommendations by providing a practical clinical perspective on identifying patients most likely to benefit from adjunctive vericiguat therapy.

## 5. Discussion

The present review integrates four complementary perspectives into a single practical clinical framework: (1) contemporary guideline recommendations and regulatory positioning, (2) mechanistic understanding of NO–sGC–cGMP pathway modulation, (3) recently published evidence from the VICTOR program and pooled analyses, and (4) a practical risk-targeted strategy for identifying patients most likely to benefit from adjunctive vericiguat therapy despite optimized GDMT.

Vericiguat occupies a distinctive place in the contemporary HF landscape because it does not seek to compete with the foundational therapies that transformed HFrEF care; rather, it addresses what those therapies may leave behind. The central clinical value of vericiguat lies in the management of residual risk—the persistent vulnerability to worsening HF, recurrent hospitalization, and progression that remains despite increasingly effective multidrug therapy.

This distinction is essential. The four pillars of HFrEF treatment have appropriately reshaped expectations regarding prognosis, but they have not eliminated the substantial burden carried by patients who continue to deteriorate despite guideline-based care. In this setting, the question is no longer whether standard therapy should be used but how best to intervene once standard therapy has already been deployed and the patient remains unstable. Vericiguat is particularly relevant in this context because it targets a biological axis—impaired NO–sGC–cGMP signaling—that is highly plausible, mechanistically coherent, and incompletely addressed by neurohormonal or sodium–glucose-directed therapies [1,2,3,12,13].

The totality of evidence suggests that vericiguat should not be interpreted through a binary framework of success or failure based on comparison with ARANI or SGLT2 inhibitors. Such a comparison, although inevitable, is conceptually incomplete. Vericiguat was never designed to replace first-line therapies that deliver the largest broad-population benefits. Its role is narrower, but that role is clinically meaningful. In high-risk patients with worsening HFrEF, even modest relative risk reduction may translate into substantial absolute benefit because event rates are high and recurrent decompensation carries profound prognostic consequences [3,10,15,22].

The VICTORIA trial established the original proof of concept in this regard. Yet the more recent VICTOR trial provides an important refinement by suggesting that the therapeutic relevance of vericiguat may extend beyond the immediate post-worsening window into a broader population of compensated but still high-risk outpatients with chronic HFrEF [6,7,8,9,10,11]. It suggests that vericiguat may be framed as a therapy for patients whose disease remains biologically and clinically unstable despite apparent outpatient compensation. The pooled participant-level analysis of VICTORIA and VICTOR is especially informative because it supports a graded, risk-based interpretation of benefit across the HFrEF continuum rather than a rigid event-defined indication [9].

A further strength of the vericiguat evidence base is the emphasis on recurrent and total HF events. This is not a trivial methodological point, as HF leads to cumulative deterioration, in which repeated admissions are not simply repeated endpoints but biological insults that accelerate myocardial, renal, and functional decline. In that light, evidence suggesting that vericiguat reduces recurrent hospitalizations and total HF events is particularly relevant [10,15]. Such an effect may be clinically more meaningful than a narrow focus on first-event time-to-event analysis would imply. Put differently, therapies that flatten the cycle of recurrent decompensation may alter the lived course of HF even when their average treatment effect appears modest on conventional summary metrics.

This drug has not demonstrated the magnitude of benefit seen with foundational therapies, nor has it emerged as a broadly effective treatment across the full spectrum of ejection fraction. The neutral experience in HFpEF, particularly in VITALITY-HFpEF, argues against extrapolation beyond the populations in which efficacy has been shown [29]. Likewise, the available data do not support describing vericiguat as a dominant symptom-relieving therapy. Its greatest strength lies in event reduction in carefully selected high-risk patients [29,42].

The safety profile, however, supports its practical integration into advanced HF care. This is highly relevant because the very patients in whom vericiguat may be most useful are also those most vulnerable to treatment-related intolerance, polypharmacy, blood pressure instability, and renal dysfunction. Reassuring data from blood pressure analyses, renal subgroup studies, nitrate interaction studies, and QTc evaluation support the view that vericiguat can be added to complex therapeutic regimens with acceptable tolerability when appropriately monitored [8,18,34,35,36].

### 5.1. Unresolved Questions and Future Directions

Despite encouraging findings from randomized clinical trials, contemporary real-world evidence for vericiguat remains relatively limited. In contrast to therapies such as SGLT2 inhibitors, whose efficacy and safety have been extensively confirmed in large observational registries and routine clinical practice, the clinical experience with vericiguat outside randomized clinical trials remains limited. Consequently, additional prospective registries and real-world studies are needed to better define treatment patterns, patient selection, long-term safety, adherence, and effectiveness across broader and more heterogeneous heart failure populations.

Several important questions remain open regarding the optimal positioning of vericiguat within contemporary heart failure management. Better phenotyping is needed to identify those patients most likely to derive disproportionate benefit from sGC stimulation. NT-proBNP is useful but unlikely to be sufficient as a standalone tool [12,13]. The role of vericiguat in HFmrEF also remains uncertain, and additional dedicated studies will be required before its use can be considered beyond HFrEF. Likewise, the optimal sequencing of vericiguat relative to other adjunctive therapies, including finerenone, has yet to be established [37,38,43,44].

Beyond these considerations, important evidence gaps remain. First, no randomized head-to-head trials have directly compared vericiguat with other adjunctive therapies used after optimization of guideline-directed medical therapy, making its relative efficacy dependent primarily on indirect comparisons and network meta-analyses. Second, although recent analyses suggest that patients with recent worsening heart failure, elevated natriuretic peptides, and persistent residual risk derive the greatest benefit, the optimal phenotypic profile for vericiguat treatment has not yet been fully established. Third, contemporary real-world registry data evaluating the effectiveness, safety, adherence, and implementation of vericiguat in routine clinical practice remain limited. Finally, long-term outcomes beyond the duration of currently available randomized clinical trials are still unknown, highlighting the need for extended follow-up studies and prospective observational registries.

Future research should extend beyond pharmacological advances to investigate how biomarker-guided strategies, phenotype-based patient selection, and AI-supported digital care models can be integrated to optimize the timing and selection of adjunctive therapies [9,22,26,27,45,46,47,48]. Integration of these approaches could facilitate earlier recognition of patients entering a worsening heart failure trajectory, improve identification of individuals most likely to benefit from vericiguat, and support more personalized, risk-targeted heart failure management [9,45,46,47,48].

### 5.2. Limitations

This review has several limitations. First, as a narrative review, it does not follow a formal systematic review methodology or PRISMA reporting framework, and no formal assessment of study quality or risk of bias was performed. Second, although every effort was made to include the most clinically relevant and up-to-date evidence, study selection was based on expert judgment and may therefore be subject to selection bias. Third, most of the currently available high-quality evidence regarding vericiguat originates from large industry-sponsored randomized clinical trials, particularly VICTORIA and VICTOR. Although these trials were rigorously designed and independently peer-reviewed, this should be considered when interpreting the overall evidence base [7,8,9,10,11]. Fourth, some of the conclusions regarding the role of vericiguat beyond worsening HFrEF rely on recently published analyses from the VICTOR program and pooled analyses, which should be interpreted cautiously because the primary endpoint of VICTOR was neutral, and several findings remain hypothesis-generating [7,8,9,10,11]. In addition, the current evidence base is limited by the absence of direct head-to-head comparisons with other adjunctive heart failure therapies, the lack of extensive real-world registry data, incomplete characterization of the optimal responder phenotype, and the absence of long-term outcome data beyond the follow-up periods of the available randomized clinical trials. Finally, the proposed risk-targeted clinical framework represents the authors’ interpretation of the currently available evidence [3,9,21,22,23,24,25,26,27] and should be considered a conceptual model intended to facilitate individualized clinical decision-making rather than a modification of existing guideline recommendations.

**Figure 2:**
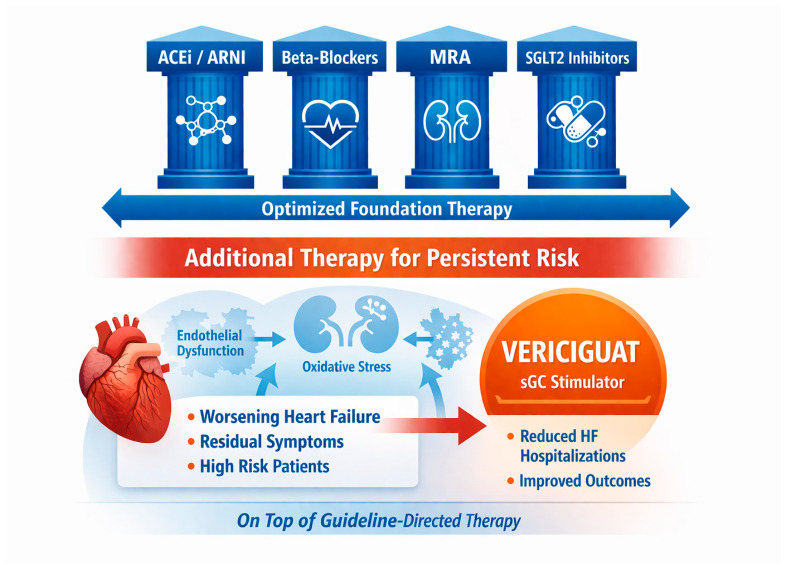
Vericiguat as an add-on therapy for persistent risk in heart failure. Vericiguat as add-on therapy in patients with heart failure who remain at high residual risk despite optimized GDMT (ACEi/ARNI, beta-blockers, MRA, and SGLT2 inhibitors). By stimulating sGC and restoring cGMP signaling impaired by endothelial dysfunction and oxidative stress, vericiguat contributes to reduced HF hospitalizations and improved clinical outcomes. The conceptual design and scientific content were created by the authors, and AI-assisted tools (ChatGPT 5, Open AI, San Francisco, CA, USA) were used solely for graphical rendering.

In sum, the contemporary interpretation of vericiguat should be neither dismissive nor inflated. It is not a fifth pillar in the sense of a universally indicated therapy with broad first-line benefit across the general HFrEF population. Yet neither is it a marginal footnote. Rather, it is a mechanistically coherent, clinically useful, and increasingly evidence-based adjunct for patients in whom worsening HF signals persistent vulnerability despite otherwise appropriate care. In that narrower but clinically important space, vericiguat may indeed represent a missing link—not in the foundation of HF treatment but in the reduction in the risk that persists after the foundation has already been laid (Figure 2).

## 6. Conclusions

Vericiguat does not challenge the primacy of the four pillars of HFrEF therapy, but it addresses what they do not fully eliminate: residual risk. In selected patients with worsening or persistently high-risk chronic HFrEF, vericiguat offers a mechanistically distinct and clinically relevant adjunctive strategy. Its role is not foundational but strategic—refining contemporary HF care where vulnerability remains greatest.

Key Messages

- Vericiguat targets impaired NO-sGC–cGMP signaling, a biologically relevant pathway not directly addressed by foundational HFrEF therapies.
- The greatest clinical value of vericiguat appears to lie in patients with worsening or persistently high-risk chronic HFrEF despite GDMT.
- Compared with ARNI and SGLT2 inhibitors, vericiguat provides incremental rather than foundational benefits.
- The VICTOR trial expanded the available evidence regarding vericiguat in ambulatory patients with chronic HFrEF but did not demonstrate superiority for its primary endpoint.
- Vericiguat may become increasingly relevant within phenotype-driven and digitally supported HF care models for identifying patients with persistent residual risk and optimizing adjunctive therapy.

## Figures and Tables

**Table 1:** Randomized clinical trials of vericiguat (*n* > 100).

Trial [Reference]	Year	Phase/ Design	Population	*N*	Intervention/ Comparator	Follow-Up	Primary Endpoint	Main Findings	Interpretation
SOCRATES-REDUCED [1]	2015	Phase II, randomized, placebo-controlled, dose-finding	Worsening chronic HFrEF after recent decompensation	456	Vericiguat (1.25–10 mg) vs. placebo	12 weeks	Change in NT-proBNP	Primary endpoint not met overall; dose–response signal with 10 mg	Proof of concept; informed phase III dose selection
SOCRATES-PRESERVED [28]	2017	Phase II, randomized, placebo-controlled	Worsening HFpEF	477	Vericiguat vs. placebo	12 weeks	NT-proBNP and LA volume	No significant effect on primary endpoints	No established role in HFpEF
VICTORIA [3]	2020	Phase III, randomized, double-blind, placebo-controlled	Chronic HFrEF with recent worsening HF	5050	Vericiguat (up to 10 mg) vs. placebo + GDMT	Median 10.8 months	CV death or first HF hospitalization	Significant reduction in primary composite endpoint (mainly HF hospitalization)	Pivotal evidence; defines indication in worsening HFrEF
VITALITY-HFpEF [29]	2020	Phase IIb, randomized, double-blind, placebo-controlled	HFpEF with recent decompensation	789	Vericiguat 10/15 mg vs. placebo	24 weeks	KCCQ physical limitation score	No improvement vs. placebo	Neutral trial; does not support HFpEF use
VICTOR [7]	2025	Phase III, randomized, double-blind, placebo-controlled	Ambulatory chronic HFrEF (no recent worsening)	6105	Vericiguat 10 mg vs. placebo + GDMT	Median 18.5 months	CV death or HF hospitalization	Primary endpoint not met; fewer CV and all-cause deaths (secondary outcomes)	Extends evidence; suggests potential mortality signal but neutral primary outcome
VICTORIA + VICTOR pooled analysis [9]	2025	Patient-level pooled analysis of RCTs	HFrEF across broader risk spectrum	11,155	Vericiguat vs. placebo	Trial-dependent	CV death or HF hospitalization	Modest reduction in pooled composite endpoint; benefit greater at lower NT-proBNP	Supports risk-based positioning

Abbreviations: CV—cardiovascular; GDMT—guideline-directed medical therapy; HFpEF—heart failure with preserved ejection fraction; HFrEF—heart failure with reduced ejection fraction; KCCQ—Kansas City Cardiomyopathy Questionnaire; LA—left atrium; NT-proBNP—N-terminal pro-B-type natriuretic peptide.

**Table 2:** Clinical checklist for vericiguat initiation and monitoring.

Step	Key Considerations	Practical Notes
Patient selection	Chronic symptomatic HFrEF with recent worsening HF despite optimized GDMT	Consider especially in patients with recent HF hospitalization, need for intravenous diuretics, recurrent decompensation, or high residual risk
Baseline assessment	Blood pressure, volume status, renal function, hemoglobin, NT-proBNP	Avoid initiation in patients with symptomatic hypotension or very low systolic blood pressure
Contraindications and situations requiring caution	Symptomatic hypotension, persistent SBP <90 mmHg, severe renal impairment (eGFR <15 mL/min/1.73 m^2^ or dialysis)	Do not initiate therapy in patients with symptomatic hypotension or persistent SBP <90 mmHg. Clinical experience in patients with eGFR <15 mL/min/1.73 m^2^ or receiving dialysis is very limited; treatment should generally be avoided in these settings
Initiation	Start with 2.5 mg once daily	Administer with food; consider only after clinical stabilization following a worsening HF event
Dose titration	Gradual up-titration to 5 mg and then 10 mg once daily as tolerated	Dose increases are usually performed at approximately 2-week intervals
Drug interactions	Concomitant vasodilator therapy, particularly nitrates	Concomitant nitrate therapy may increase the risk of symptomatic hypotension. Blood pressure should be monitored carefully, particularly in patients receiving additional vasodilators or multiple antihypertensive agents.
Early follow-up	Reassess blood pressure, symptoms of hypotension, volume status, and tolerability after initiation or dose change	Closer monitoring is advisable in patients with borderline blood pressure, advanced HF, renal impairment, or polypharmacy
Ongoing monitoring	Periodic assessment of blood pressure, hemoglobin, renal function, congestion status, and HF events	Monitoring can be integrated into routine HF follow-up; vigilance for anemia and hypotension is recommended
Management of hypotension	Evaluate volume depletion, diuretic intensity, and concomitant vasodilators	Temporary dose reduction or interruption may be considered if clinically needed
Management of anemia	Monitor hemoglobin, particularly in patients with baseline anemia or frailty	Clinically significant anemia should prompt evaluation for alternative causes
Therapeutic positioning	Add-on therapy for selected high-risk HFrEF patients despite foundational GDMT	Not a replacement for ARNI/ACEi/ARB, beta-blockers, MRA, or SGLT2 inhibitors; best viewed as a risk-targeted adjunct

Abbreviations: ACEi—angiotensin-converting enzyme inhibitor; ARB—angiotensin receptor blocker; ARNI—angiotensin receptor–neprilysin inhibitor; eGFR—estimated glomerular filtration rate; GDMT—guideline-directed medical therapy; HF—heart failure; HFrEF—heart failure with reduced ejection fraction; MRA—mineralocorticoid receptor antagonist; NT-proBNP—N-terminal pro–B-type natriuretic peptide; SGLT2—sodium–glucose cotransporter 2.

**Table 3:** Comparison of guideline recommendations, regulatory indications, clinical evidence, and the proposed risk-targeted positioning of vericiguat.

Aspect	Current Evidence and Proposed Positioning
Mechanistic rationale	Restoration of impaired NO–sGC–cGMP signaling to complement GDMT
Regulatory indication	Symptomatic chronic HFrEF following recent worsening HF requiring hospitalization or intravenous diuretic therapy
Current guideline recommendation	Consider in selected high-risk patients despite optimized GDMT
Pivotal supporting evidence	SOCRATES-REDUCED: biological proof-of-concept; VICTORIA: reduction in CV death/HF hospitalization (mainly HF hospitalization); VICTOR: neutral primary outcome with hypothesis-generating secondary findings
Patients most likely to benefit	Recent worsening HF, recurrent hospitalization, persistent congestion, elevated NT-proBNP, evidence of residual clinical risk
Not intended for	Stable low-risk HFrEF or replacement of foundational GDMT
Authors’ proposed framework	Risk-targeted adjunctive therapy guided by residual biological and clinical risk, complementing guideline-directed foundational therapy

Abbreviations: CV—cardiovascular; GDMT—guideline-directed medical therapy; HF—heart failure; HFrEF—heart failure with reduced ejection fraction; NO–sGC–cGMP—nitric oxide–soluble guanylate cyclase–cyclic guanosine monophosphate; NT-proBNP—N-terminal prohormone of brain natriuretic peptide.

## Data Availability

No new data were created or analyzed in this study.

## Abbreviations

The following abbreviations are used in this manuscript:

ACEi	angiotensin-converting-enzyme inhibitors
ARB	angiotensin II receptor blockers
ARNI	angiotensin receptor/neprilysin inhibitor
cGMP	cyclic guanosine monophosphate
GDMT	guideline-directed medical therapy
HF	heart failure
HFmrEF	heart failure with mildly reduced ejection fraction
HFpEF	heart failure with preserved ejection fraction
HFrEF	heart failure with reduced ejection fraction
LA	left atrium
KCCQ	Kansas City Cardiomyopathy Questionnaire
MRA	mineralocorticoid receptor antagonist
NO–sGC–cGMP	nitric oxide–soluble guanylate cyclase–cyclic guanosine monophosphate
NT-proBNP	N-terminal prohormone of brain natriuretic peptide
sGC	soluble guanylate cyclase
SGLT2	sodium–glucose cotransporter 2
